# Risk factors for postoperative haemorrhage after total thyroidectomy: clinical results based on 2,678 patients

**DOI:** 10.1038/s41598-017-07334-1

**Published:** 2017-08-01

**Authors:** Xu Zhang, Wei Du, Qigen Fang

**Affiliations:** Department of Head Neck and Thyroid Surgery, Affiliated Cancer Hospital of Zhengzhou University, Henan Cancer Hospital, Zhengzhou, Henan Province PR China

## Abstract

The aim of this study was to analyse postoperative haemorrhage (POH) after a total thyroidectomy and explore the possible risk factors. Records of patients receiving a total thyroidectomy were reviewed and analysed for risk factors of POH. From the 2,678 patients in this study, a total of 39 patients had POH, representing an incidence of 1.5%. The majority (59.0%) of POH events occurred within four hours after surgery. Arterial haemorrhage was the primary cause of POH and was identifiable prior to venous bleeding, making it the first sign of POH. A univariate analysis revealed an association between POH, certain disease factors and BMI, but only a BMI greater than 30 was found to significantly increase the risk of POH (almost 6-fold). At the first sign of POH, all patients showed an obvious red drainage, and 92.3% of the patients had neck swelling. In summary, arterial bleeding is the main cause and first sign of postoperative haemorrhage, as it starts earlier than venous bleeding. A BMI greater than 30 significantly increases the risk of neck haematoma.

## Introduction

A large proportion of the global increase in thyroid cancer is thought to be due to a real increase in the incidence and/or increased detection of papillary thyroid cancer^1^. Surgery is the preferred procedure for head and neck surgeons. Total thyroidectomy has been regarded as a safe and effective operative option for selected patients^2, 3^.

Postoperative haemorrhage (POH) is an uncommon complication after thyroid surgery. Although some haematomas present superficially, in severe cases, haematomas can be lethal if they result in airway compression. Occasionally, blood transfusions are required. Therefore, patients undergoing thyroidectomy need close postoperative monitoring for any signs of bleeding. Various studies have reported the incidence of POH, which ranges from 0 to 4.2%^4–13^. Three categories of risk, namely, surgical technique, patient predisposition, and thyroid pathology, are responsible. Weiss *et al*.^4^ has determined that high-volume hospitals and the female sex are important for decreased haematoma risk, and partial thyroidectomy, inflammatory thyroid disease, bleeding disorders, and chronic kidney disease increase the risk of haematoma. Recently, Liu *et al*.^5^ reported that the occurrence of POH is just 0.85% and that benign pathology and a previous thyroid history are individual risk factors. Chavez *et al*.^13^ notes an interesting finding that in a thyroid operation, compared to the traditional tie and suture technique, the use of an advanced bipolar device reduces the operation time with a similar postoperative outcome profile. All of these studies used a range of different surgical procedures, including partial thyroidectomy, unilateral lobectomy and subtotal thyroidectomy, but few authors have focused on the association between POH and total thyroidectomy^4–13^. Therefore, in this study, we analysed POH after a total thyroidectomy and explored the possible risk factors.

## Results

From a total of 2,678 patients, 39 (15 male and 24 female) had POH, which was equal to an incidence of 1.5%. The mean age was 56.5 (range: 44–67) years old. Four patients received a bilateral cervical dissection (regions II–V), and 10 patients received a unilateral cervical dissection (regions II–V). All patients were diagnosed with papillary thyroid cancer.

POH was detected at various times post-surgery: in 15 (38.4%) patients, the first sign of POH occurred within 2 hours after surgery; in 8 (20.5%) patients, the first sign of POH occurred between 2 to 4 hours after surgery; and in 10 (25.6%) patients, the first sign of POH occurred between 4 to 8 hours after surgery. POH was not first detected more than 12 hours after surgery.

In 23 (59.0%) patients, the cause of POH was recognized as arterial bleeding: a branch of the superior thyroid artery, 15 cases; a branch of the inferior thyroid artery, 7 cases; and a branch of the transverse cervical artery, 1 case. In 13 (33.3%) patients, the cause of POH was recognized as venous bleeding: the anterior jugular vein, 4 cases; the inferior thyroid vein, 3 cases; a branch of the internal jugular vein, 2 cases; a subfascial vein, 1 case; subcutaneous tissue, 2 cases; and the remnant thyroid tissue of the Zuch nodule, 1 case. In the remaining three (7.7%) patients, no apparent bleeding source was noted, and there was no obvious blood clot during the exploratory operation (Table 1).

**Table 1:** Summary of bleeding sources.

Category	Location	n
Artery (n = 23)	Branch of superior thyroid artery	15
	Branch of inferior thyroid artery	7
	Branch of transverse cervical artery	1
Venous (n = 13)	Anterior jugular vein	4
	Inferior thyroid vein	3
	Branch of internal jugular vein	2
	Subfascial vein	1
	Subcutaneous tissue	2
	Remnant thyroid tissue of Zach nodule	1
Unknown (n = 3)		3

In patients with arterial bleeding, the first sign of POH was noted in 12 patients within two hours after surgery, six patients between two and four hours after surgery and in five patients between four and eight hours after surgery. In patients with venous bleeding, the first sign of POH was noted in three patients within two hours after surgery, two patients between two and four hours after surgery, five patients between four and eight hours after surgery, and three patients between eight and 12 hours after surgery. The difference was significant (p = 0.039) (Table 2).

**Table 2:** Compare of different time of the first sign of artery or venous bleeding.

	Artery (n = 23)	Venous (n = 13)	p
0–2 hours	12	3	0.039
2–4 hours	6	2	
4–8 hours	5	5	
8–12 hours	0	3	

All patients with POH had malignant thyroid diseases, compared to 87.4% in patients without POH; the difference was significant (p = 0.023). The majority (79.5%) of patients with POH had a BMI greater than 30, compared to 12.8% of patients without POH; the difference was significant (p < 0.001). A univariate analysis showed that there were no other potential risk factors (all p > 0.05). Therefore, a multivariate analysis revealed that a BMI greater than 30 would increase the risk of POH nearly 6-fold (OR: 6.002, p = 0.023) (Table 3).

**Table 3:** Risk factor analysis of POH*.

		Patients with POH	Patients without POH	Univariate	Multivariate
Sex	Female	24	1825		
	Male	15	814	0.307	
Age	<55	18	1213		
	>=55	21	1426	0.981	
Disease	Benign	0	333		
	Malignant	39	2306	0.023	0.124
LND^#^	Yes	14	898		
	No	25	1741	0.807	
Diabetes	Yes	4	312		
	No	35	2327	0.814	
Hypertension	Yes	10	612		
	No	29	2027	0.719	
Antiplatelet	Yes	1	136		
	No	38	2503	0.720	
BMI^&^	>30	31	337		
	<30	8	2302	<0.001	0.031
Smoker	Yes	8	473		
	No	31	2166	0.676	

^*^POH: Postoperative hematoma; ^#^LND: Lateral neck dissection; ^&^BMI: Body Mass Index.

To determine how the cancer subtype and disease stage (based on the 2016 ATA guidelines) affected the POH, patients with malignant tumours were separately analysed, and it was noted that cancer subtype, tumour stage, node stage, and disease stage were not associated with POH (all p > 0.05, Table 4).

**Table 4:** Risk factor analysis of POH* in patients with malignant tumor.

	Patients with POH	Patients without POH	Univariate
Cancer subtype			
papillary thyroid carcinoma	39	2265	
medullary thyroid carcinoma	0	26	
follicular thyroid carcinoma	0	15	1.000
Tumor stage			
T1	21	1012	
T2	12	961	
T3	5	295	
T4	1	38	0.523
Node stage			
N0	17	985	
N1	22	1321	1.000
Disease stage			
I	8	626	
II	13	689	
III	14	822	
IV	4	169	0.757

^*^POH: Postoperative hematoma.

At the first sign of POH, all patients had an obvious red drainage, 92.3% of the patients had apparent neck swelling and 12.8% of the patients had bleeding from the incision. Four (10.26%) patients whose causes of POH were recognized as arterial bleeding complained of dyspnoea. No patients underwent an emergency tracheotomy or a third operation. No patients died of POH.

## Discussion

This study is the first to report that arterial bleeding allows for earlier identification of POH, as arterial bleeding was discovered prior to venous bleeding. This finding is important because it may allow surgeons to plan for more targeted surgeries in exploratory operations. The source of the haematoma could almost always be found (92.3%), and the majority of haematomas were caused by arterial bleeding. This finding is consistent with other previous studies^5, 7^. Moreover, it was noted that the distribution of venous bleeding was more complex, making it more difficult to isolate the site of origin when venous bleeding was suspected.

The role of BMI in surgery-related complications has been well studied. Desai *et al*.^16^ found that rates of reoperation and skin necrosis were significantly lower in patients who had undergone complex abdominal wall reconstructions if the patients had a BMI lower than 30, compared to subgroups with a greater BMI. During surgical resections of intracranial tumours, Wei *et al*.^17^ noted that BMI was closely linked to preoperative-to-postoperative plasma fibrinogen consumption, and postoperative fibrinogen deficiency was a potential risk for postoperative bleeding. Similarly, Ahmed *et al*.^18^ reported that BMI was an independent risk factor for vascular complications in patients undergoing cardiac catheterization. Moreover, BMI has been associated variably with head and neck cancer outcomes. Gama *et al*.^19^ found that being underweight at diagnosis was an independent, adverse prognostic factor, whereas being overweight conferred a better prognosis. In a study aiming to analyse how BMI affects infectious complications in free tissue transfer surgery^20^, the authors failed to note a positive association, and a similar finding was also reported by de la Garza *et al*.^21^. However, both Lo *et al*.^22^ and Heo *et al*.^23^ concluded that a poor nutrition status, indicated by low BMI, was associated with a greater tendency to develop postoperative complications in free tissue transfer for head and neck reconstruction. However, few authors have analysed the association between BMI and POH in thyroid surgery. This study discovered that a BMI greater than 30 would increase the risk of POH nearly 6-fold. An important interpretation is that a deficiency in neck extension in obese patients leads to limited exposure of the surgical field and increases operation difficulty.

The incidence of POH in this study was 1.5%, and this finding was consistent with previous research^4, 5^. However, in this study, all patients underwent a total thyroidectomy, and a more extensive thyroid resection tends to increase the POH risk^7, 9^. Promberger *et al*.^7^ noted that, compared with a subtotal bilateral resection, a bilateral near-total resection and total thyroidectomy results in a significantly higher risk of POH. Godballe *et al*.^9^ reported that bilateral thyroid surgery had a significant influence on the frequency of POH. Possible explanations for our findings might be that all the procedures were performed by extremely experienced surgeons, so the risk of POH was reduced^6^.

Complications from emergency tracheotomy or death resulting from POH are extremely rare, and neither we nor others have reported such serious events^8, 10, 12^. A previous study showed that the mean time between skin closure and the first sign of POH was threefold longer in patients undergoing emergency tracheotomy relative to patients without an emergency tracheotomy^7^. Most of the POH events were noted within four hours after surgery in our study. At the first sign of POH, all patients had an obvious red drainage, and 92.3% of the patients had apparent neck swelling; these signs might be reliable indicators of POH. A timely exploratory operation was strongly suggested when there were such signs. Overly conservative treatment might lead to serious complications.

A number of studies have evaluated the effects of blood pressure on the incidence of POH. Morton *et al*.^8^ concluded that a post-anaesthetic systolic blood pressure in excess of 150 mmHg significantly increased the risk of haemorrhage following thyroid surgery. In another study, the authors reported a similar finding^11^. We agree, even though hypertension was not associated with POH in our study. In our cancer centre, patients with POH usually (more than 90%) had severe nausea and vomiting before the symptoms of POH emerged (unpublished data), during which the systolic blood pressure was more than 150 mmHg. Therefore, routine postoperative inhibition of vomiting might be helpful for reducing the number of POH events.

A conflicting result was described regarding the association between POH and disease pathology^4–13^. It could be explained by a range of different surgical procedures and various disease categories, including Graves’ disease. No POH event was noted in patients with benign disease in the current study, and this observation might be well attributed by the following reasons. Firstly, Graves’ disease was not treated at our institute. Secondly, benign disease usually has a clear boundary and can easily be dissected. Thirdly, total thyroidectomy was the only surgical procedure, and the resection extent was significantly associated with neck haematoma^4, 7^.

In summary, postoperative haemorrhage is uncommon following total thyroidectomy. Arterial bleeding is the primary cause and is the first sign of postoperative haemorrhage, as it occurs earlier than venous bleeding. Obvious red drainage and apparent neck swelling are reliable indicators of postoperative haemorrhage. A timely exploratory operation is suggested to avoid severe complications. A BMI greater than 30 significantly increases the risk of neck haematoma.

## Methods

The Zhengzhou University institutional research committee approved our study, and all participants signed an informed consent agreement. All methods were performed in accordance with the relevant guidelines and regulations.

From May 2014 to October 2016, after filtering patients who had undergone previous neck or thyroid surgery, we reviewed a consecutive series of 2,678 patients (829 male and 1,849 female) who had received a total thyroidectomy in the Department of Head, Neck and Thyroid Surgery at the Affiliated Cancer Hospital of Zhengzhou University. All related information including age, sex, smoking history, BMI, surgical records, postoperative records, pathology reports, time between skin closure and the first sign of POH and other related factors was obtained. A total of 2,345 patients had malignant thyroid diseases, and 316 patients underwent bilateral or unilateral neck dissection. A total of 137 patients had previously taken antiplatelet drugs routinely, and they were required to stop at least 1 week before the operation. A total 481 patients were recognized as current smokers. Smokers were defined as patients who were current smokers or had quit smoking for no more than half a year^14^. A total of 368 patients were obese according to the WHO guideline (BMI > 30). Patients who had POH had undergone secondary surgical interventions.

The collar incision was performed after the patient received general balanced anaesthesia. The strap muscles were divided in the middle and retracted laterally, and the subcutaneous tissue and platysma muscle were subsequently divided. The superior, middle, and inferior thyroid vessels were ligated with knot tying regardless of vessel diameter. All thyroid glands were resected using the capsular principle of dissection^15^. Routine applications of haemostatic agents and operative bed drainage prior to closure was performed.

General data were analysed by means of a chi-squared test or Fisher exact test. Independent risk factors were analysed by multivariable logistic regression. All statistical analyses were performed using SPSS 13.0. A p < 0.05 was considered significant.

### Data availability

The corresponding author is responsible for the data availability.
